# In vitro evaluation of the monoterpene linalool and the nematophagous fungus *Arthrobotrys cladodes*, individually and in combination, against gastrointestinal nematodes of sheep

**DOI:** 10.1007/s11250-026-05168-6

**Published:** 2026-06-22

**Authors:** Antônia Aniellen Raianne Moisés Aguiar, Ana Maria Santos Lima, Fabio Ribeiro Braga, Jackson Victor Araújo, Thais Ferreira Feitosa, Vinícius Longo Ribeiro Vilela

**Affiliations:** 1 Postgraduate Program in Science and Animal Health, Federal University of Campina Grande, State of Paraíba, Patos, Brazil; 2 Department of Veterinary Medicine, Federal Institute of Paraíba, State of Paraíba, Sousa, Brazil; 3 Experimental Parasitology and Biological Control Laboratory, University Vila Velha, Vila Velha State of Espírito Santo, Brazil; 4 Department of Veterinary Medicine, Federal University of Viçosa, Viçosa, State of Minas Gerais Brazil

**Keywords:** Biological control, Integrated control, Phytotherapy, Helminths, Small ruminants, Anthelmintic resistance

## Abstract

Anthelmintic resistance has prompted the search for alternative control strategies, such as the use of plant-derived compounds and biological control with nematophagous fungi. The aim of the present study was to evaluate the in vitro anthelmintic effect of linalool and its association with the fungus *Arthrobotrys cladodes* on gastrointestinal nematodes of sheep. The larvicidal activity of linalool, alone and in combination with *A. cladodes* conidia, was assessed using the Larval Motility Inhibition Test (LMIT) on infective larvae (L3). Ten (10) experimental groups were designed, each with eight replicates, comprising treatments with linalool and *A. cladodes* conidia, alone and in combination, as well as ivermectin (positive control) and distilled water (negative control). Larval motility was evaluated under optical microscopy after 1, 7, and 14 days of exposure. A significant reduction (*p* ≤ 0.05) in the number of L3 larvae was observed in the groups treated with linalool combined with *A. cladodes* (G7–G10) compared to the negative control group (G1), starting at 24 h and reaching 100% reduction after 14 days. The groups treated only with *A. cladodes* conidia (G3 and G4) showed less effective reductions compared to the combined treatments. The larvicidal activity of the groups treated solely with linalool (G5 and G6) did not differ statistically (*p* > 0.05) from those treated with the fungus and linalool. It is concluded that the combination of linalool and *A. cladodes* conidia exhibits anthelmintic activity against gastrointestinal nematodes of sheep, demonstrating a synergistic interaction between the nematophagous fungus and the monoterpene.

Gastrointestinal nematode infections remain one of the main challenges to the productive advancement of sheep farming, compromising animal health and welfare, restricting productivity, and leading to significant economic losses (Sebai et al. 2022; Štrbac et al. 2023). The use of anthelmintic drugs—traditionally employed for the control of gastrointestinal nematodes—has had its efficacy compromised due to the intensive and improper administration of these compounds, favoring the selection and development of resistant parasite populations (Zajícková et al. 2020).

As a result, there is an increasing search for new treatment strategies that are effective in controlling infections, that reduce dependence on chemical products, and that are economically sustainable (Vilela et al. 2020; Castagna et al. 2022; Sebai et al. 2022). Among the promising alternative strategies, biological control using nematophagous fungi and phytotherapy employing plant-derived compounds with anthelmintic activity have attracted growing interest (Helal et al. 2020; Rodrigues et al. 2020; Štrbac et al. 2023; Ribeiro and Vilela 2023).

Nematophagous fungi represent an effective and sustainable alternative, as they act by attacking the infective forms of parasites present in pastures without causing environmental damage (Vieira et al. 2019; Li et al. 2022). *Arthrobotrys cladodes* is a predatory fungus widely studied for the control of gastrointestinal infections in different animal species (Oliveira et al. 2018; Vieira et al. 2019, 2020a; Rodrigues et al. 2020), notable for possessing desirable traits for nematophagous agents, such as a high capacity for conidia production and the formation of chlamydospores, which favor its dispersal and environmental colonization, thus enhancing its potential as a biocontrol tool (Oliveira et al. 2018).

Plant-derived products have emerged as a promising alternative to synthetic drugs, as they contain bioactive compounds with anthelmintic properties, such as monoterpenes. Linalool is a volatile compound originating from plant secondary metabolism and an important component of the essential oils of several aromatic plant species (Saeed et al. 2023). This monoterpene exhibits multiple biological properties, including antiparasitic activity, which has sparked growing interest as an alternative for the control of gastrointestinal nematodes (Katiki et al. 2017; Helal et al. 2020, 2022; Alvares et al. 2025).

The use of a single control method for helminth infections has proven unsustainable and economically ineffective (Maqbool et al. 2017; Simões et al. 2022; Štrbac et al. 2023). Therefore, the adoption of Integrated Parasite Control (IPC) practices, which combine multiple strategic measures, emerges as a viable approach to reduce pasture contamination, infection intensity in animals, and dependence on anthelmintic drugs, while maintaining their efficacy—particularly in cases of resistance (Costa et al. 2011; Simões et al. 2022).

The combination of *A. cladodes* with other fungal species has shown promising results in the control of gastrointestinal nematodes, as the interaction of different mechanisms of action may promote synergistic and complementary biological control effects (Vieira et al. 2019, 2020b). Similarly, significant anthelmintic activities have been observed with the association of plant-derived compounds against nematodes of small ruminants (Katiki et al. 2017, 2019; Helal et al. 2020). However, studies evaluating the association of nematophagous fungi with bioactive plant-derived compounds, such as monoterpenes, remain scarce. Therefore, the aim of the present study was to evaluate the in vitro anthelmintic effect of linalool and its association with the fungus *A. cladodes* in the control of gastrointestinal nematodes of sheep.

For the experiment, L3 larvae of gastrointestinal nematodes were obtained from coprocultures (Roberts and O’Sullivan 1950) prepared using fecal samples from a flock of approximately 60 naturally infected Santa Inês sheep belonging to the Federal Institute of Paraíba (IFPB), Sousa campus. Infection was confirmed by fecal egg count (eggs per gram of feces – EPG) (Gordon and Whitlock 1939). L3 larvae were recovered using a modified Baermann technique (Willcox and Coura 1989), and the nematode population was identified according to the criteria described by Taylor et al. (2017), consisting of *Haemonchus* sp. (86%), *Trichostrongylus* spp. (10%), and *Oesophagostomum* sp. (4%).

The nematophagous fungus *A. cladodes* (isolate CG 719) was obtained from soil samples from the Zona da Mata region, state of Minas Gerais, Brazil. The isolate was maintained at 4 °C in the dark in culture tubes containing 2% corn meal agar (CMA 2%) in the mycological collection of the Department of Veterinary Medicine, Federal University of Viçosa. For fungal culture, small portions of the isolate were transferred to 9 cm Petri dishes containing 2% potato dextrose agar (PDA) and incubated for 10 days in the dark. After this period, 10 mL of distilled water was added to each Petri dish to obtain the conidial suspension. To facilitate the release of conidia, the culture surface was gently brushed with a fine paintbrush. The resulting suspension was collected with a Pasteur pipette and transferred to 15 mL Falcon tubes. Conidial concentration was determined using a Neubauer chamber.

The Larval Motility Inhibition Test (LMIT) was performed to evaluate the effect of *A. cladodes* in association with linalool on the motility inhibition of L3 larvae of gastrointestinal nematodes of sheep, following the methodology described by Ferreira et al. (2013). The monoterpene linalool (≥ 97% purity) (Sigma Chemical Co., St. Louis, MO, USA) and the anthelmintic ivermectin (Sigma Chemical Co., St. Louis, MO, USA), technical grade, were used. For the assay, a concentration containing approximately 130 L3/250 µL was obtained from coprocultures.

Ten experimental groups were established in 1.5 mL microtubes, with a final volume of 500 µL, each containing 250 µL of the larval suspension (130 L3) incubated with an equal volume of the treatment solution (Table 1). The lethal doses to inhibit 25% (LD25) and 50% (LD50) of the larval population with linalool and ivermectin were previously determined by Aguiar et al. (2025) in a study using the same nematode population. For linalool, LD25 was 1.5 mg/mL (95% CI = 0.08–0.23) and LD50 was 4.2 mg/mL (95% CI = 0.29–0.57). For ivermectin, LD50 was 0.53 mg/mL (95% CI = 0.38–0.67), used as the positive control group (G2).

**Table 1:** Experimental groups and composition of the treatments with linalool and *Arthrobotrys cladodes* (CG 719), evaluated individually or in combination, against infective larvae of gastrointestinal nematodes of sheep

Experimental groups	Composition
Group 1 (negative control)	250 µL (130 L3) + 250 µL (destilled water)
Group 2 (positive control)	250 µL (130 L3) + 250 µL (DL50 ivermectin)
Group 3	250 µL (130 L3) + 250 µL (10.000 conidia)
Group 4	250 µL (130 L3) + 250 µL (100.000 conidia)
Group 5	250 µL (130 L3) + 250 µL (DL25 linalool)
Group 6	250 µL (130 L3) + 250 µL (DL50 linalool)
Group 7	250 µL (130 L3) + 250 µL (10.000 conidia + DL25 linalool)
Group 8	250 µL (130 L3) + 250 µL (10.000 conidia + DL50 linalool)
Group 9	250 µL (130 L3) + 250 µL (100.000 conidia + DL25 linalool)
Group 10	250 µL (130 L3) + 250 µL (100.000 conidia + DL50 linalool)

Each experimental group was prepared in eight replicates and incubated in a biochemical oxygen demand (BOD) incubator at 27–28 °C and 85–90% relative humidity. After 1 (24 h), 7, and 14 days of exposure to the treatments, larval motility was evaluated under optical microscopy (100× magnification), quantifying larvae as motile (alive) or immobile (dead). The percentage of larval reduction was calculated as a parameter to estimate the inhibitory efficacy of the tested treatments, based on larval motility. This indicator represents the reduction in the number of live (motile) infective larvae (L3) in the treated groups compared with the negative control group. Considering that motility is directly associated with larval viability and infective potential, a decrease in the number of motile larvae reflects an inhibitory effect of the tested compounds, with immobile larvae being considered dead for the calculation of inhibitory efficacy. The inhibition efficacy of L3 motility was determined using the following formula:

$${\%}\:\mathrm{L}\mathrm{a}\mathrm{r}\mathrm{v}\mathrm{a}\mathrm{l}\:\mathrm{r}\mathrm{e}\mathrm{d}\mathrm{u}\mathrm{c}\mathrm{t}\mathrm{i}\mathrm{o}\mathrm{n}=\frac{\mathrm{M}\mathrm{e}\mathrm{a}\mathrm{n}\:\mathrm{o}\mathrm{f}\:\mathrm{L}3\:\mathrm{i}\mathrm{n}\:\mathrm{t}\mathrm{h}\mathrm{e}\:\mathrm{N}\mathrm{C}\mathrm{G}-\mathrm{M}\mathrm{e}\mathrm{a}\mathrm{n}\:\mathrm{o}\mathrm{f}\:\mathrm{L}3\:\mathrm{i}\mathrm{n}\:\mathrm{t}\mathrm{h}\mathrm{e}\:\mathrm{T}\mathrm{G}\:}{\begin{array}{c}\text{Mean of L3 in the NCG}\\\:\:\end{array}}\times\:100$$

Where: NCG: Negative Control Group; TG: Treated Group.

The raw data obtained in the assays were initially assessed for normality. After confirmation of this assumption, a one-way Analysis of Variance (ANOVA) was performed, followed by Tukey’s test at a 5% significance level. Tukey’s test was adopted due to the nature of the comparisons, which involved balanced groups and homogeneous variances, making it appropriate for multiple comparisons among experimental groups, with control of Type I error and ensuring the robustness of the results. All statistical analyses and normality tests were performed using GraphPad Prism software (version 8.0).

After the first day of interaction, there was a significant reduction (*p* ≤ 0.05) in the number of infective larvae in the treated groups compared to the negative control (Table 2). Linalool, both alone and in combination with the fungus *A. cladodes*, exhibited significant anthelmintic activity by inhibiting the motility of infective larvae. When tested alone, linalool showed inhibitory activity on L3 motility, with an average larval reduction of 60.2% (G5—LD25) and 78.8% (G6—LD50) after one day, reaching 100% reduction after 14 days (D14). These findings corroborate those of Aguiar et al. (2025), who also observed a marked reduction in L3 motility of 41.5% (1.5 mg/mL) and 72.8% (4.2 mg/mL) after 24 h of exposure to linalool, and 100% after 14 days. These results demonstrate that this biocompound exhibits a rapid larvicidal action with a prolonged inhibitory effect, resulting in high larval reduction percentages at the end of the experimental period. Other studies have also reported the rapid inhibitory action of linalool on the motility of infective larvae of gastrointestinal nematodes, showing significant reductions after 24 h of exposure to this monoterpene (Helal et al. 2020, 2022).

**Table 2:** Mean number of live larvae ± standard deviation and percentages of reduction of infective larvae (motility inhibition) of gastrointestinal nematodes of sheep after 1 (24 h), 7, and 14 days of incubation

Experimental groups	Time after treatment					
	Day 1 (24 h)		Day 7		Day 14	
	Mean ± SD	R (%)	Mean ± SD	R (%)	Mean ± SD	R (%)
Group 1	130^A^ ± 20.2	-	130^A^ ± 21.8	-	130^A^ ± 5	-
Group 2	103.3^A^ ± 2.9	20.5	0^C^ ± 0	100	0^C^ ± 0	100
Group 3	108.3^A^ ± 37.9	16.7	74^B^ ± 17.4	43	43.3^B^ ± 2,9	66.7
Group 4	70^B^ ± 22.9	45.2	60^B^ ± 10	53.8	25^B^ ± 5	80.8
Group 5	51.7^B^ ± 10.4	60.2	16.7^C^ ± 16.1	87.2	0^C^ ± 0	100
Group 6	28.3^C^ ± 10.4	78.2	5^C^ ± 0	96.2	0^C^ ± 0	100
Group 7	33.3^C^ ± 7.6	74.4	3.3^C^ ± 2.9	97.5	0^C^ ± 0	100
Group 8	25^C^ ± 5	80.8	0^C^ ± 0	100	0^C^ ± 0	100
Group 9	33.3^C^ ± 7.6	74.4	5^C^ ± 5	96.2	0^C^ ± 0	100
Group 10	16.7^C^ ± 2.9	87.2	0^C^ ± 0	100	0^C^ ± 0	100

SD: Standard deviation; R (%): Percentage of reduction (motility inhibition). Means followed by different letters within the same column indicate statistically significant differences (*p* ≤ 0.05) among groups within the same evaluation period, according to Tukey’s test at a 5% probability level

The groups treated with *A. cladodes* conidia in combination with linalool (G7–G10) also demonstrated inhibitory activity on larval motility from 24 h onward, with high reduction percentages that increased over time and were significantly more effective (*p* ≤ 0.05) compared to the use of conidia alone (G3 and G4). These combined treatments achieved 100% larval reduction after 14 days of interaction, showing results similar to the positive control (G2 – Ivermectin), with no significant statistical difference (*p* > 0.05). The greater effectiveness of the combined treatment may be attributed to a synergistic interaction between the nematophagous fungus and the monoterpene, which enhanced larvicidal efficacy through the integration of distinct mechanisms of action.

In addition to the individual efficacy reported for *A. cladodes*, its combination with other fungal species has also been widely investigated and has shown promising results (Rodrigues et al. 2020; Vieira et al. 2019, 2020a), as the joint use of multiple nematophagous fungi may minimize potential limitations of individual administration and even potentiate their effectiveness as biocontrol agents (Luns et al. 2018). The findings of the present study are consistent with those of Aguiar et al. (2025), who also observed a greater reduction in L3 larvae of ovine nematodes when combining a nematophagous fungus, *Duddingtonia flagrans*, with linalool, without compromising the fungal predatory capacity. These results reinforce the enhanced anthelmintic effect achieved through the combination of biological agents, suggesting a synergistic interaction.

In the groups composed of conidia—both alone (G3 and G4) and in association with linalool (G7–G10)—trap formation and predation of infective larvae were observed, confirming the predatory activity of the nematophagous fungus. Contrary to reports in the literature, where linalool has been described as having antifungal properties (Li et al. 2023; Medeiros et al. 2022), the predatory activity of *A. cladodes* was not affected. This was confirmed in the groups associated with the monoterpene (G7–G10) by the presence of traps and the fungus’s ability to capture larvae, demonstrating that no antagonistic effect occurred.

No significant differences (*p* > 0.05) were observed between the larvicidal activity of the groups treated with the combination of linalool and *A. cladodes* conidia (G7 to G10) and those treated exclusively with linalool (G5 and G6) under the in vitro conditions evaluated. Although no differences were detected in the larvicidal activity among these groups, the combined use of the conidia and the monoterpene may offer practical and environmental advantages compared with isolated treatments, as it integrates distinct and complementary modes of action. However, it is important to emphasize that the experimental conditions adopted do not fully reproduce the biological, physiological, and environmental conditions present in in vivo systems. Nevertheless, the association between linalool and *A. cladodes* may still represent a promising approach, as linalool exerts a direct larvicidal effect on parasitic stages present in the host, whereas *A. cladodes* acts in the environment through the predation of infective larvae. The complementarity of these modes of action reinforces the potential and feasibility of this association, enhancing treatment effectiveness and contributing to sustainable integrated control strategies.

Although the mechanism of action of linalool has not been fully elucidated, evidence indicates that it mainly affects parasite motility and viability (Helal et al. 2020, 2022; Aguiar et al. 2025). It is suggested that its activity is related both to disruption of membrane function through strong lipolytic activity and to neurotoxic effects similar to those of organophosphates, inhibiting acetylcholinesterase (AChE) receptors, which leads to acetylcholine accumulation and neuromuscular overstimulation, resulting in nematode paralysis and death (López and Pascual-Villalobos 2010; Nadeem 2013; Adeyinka et al. 2023). In contrast, the mechanism of action of *A. cladodes* involves the formation of specialized three-dimensional adhesive hyphal traps that promote adhesion, immobilization, penetration, and destruction of infective larvae in the environment (Oliveira et al. 2018). This predatory activity disrupts the nematode life cycle, reducing the availability of infective larvae on pasture and, consequently, lowering the reinfection rate in animals (Vilela et al. 2018; Rodrigues et al. 2022).

The control of helminth infections in sheep has been improved through the adoption of integrated sustainable practices in which biological agents and natural compounds emerge as promising alternatives, either in combination or as substitutes for conventional drugs (Maqbool et al. 2017; Burke and Miller 2020; Mendes et al. 2023). Despite advances in studies evaluating combined control methods, research investigating the association between nematophagous fungi and plant-derived compounds remains scarce, although evidence already suggests that such interactions may result in synergistic effects (Aguiar et al. 2025). In this context, the present study was the first to evaluate the efficacy of combining the monoterpene linalool with *A. cladodes* as an innovative proposal for the control of gastrointestinal nematodes in sheep.

The association of *A. cladodes* conidia with linalool also produced results comparable to the group treated with the anthelmintic ivermectin (G2) after 14 days, with no significant statistical difference (*p* > 0.05), demonstrating that the combination achieved efficacy levels similar to the drug. In the current scenario, where anthelmintic resistance to major chemical classes of commercial drugs, including ivermectin, represents one of the greatest challenges in global sheep production (Gilleard et al. 2021), these findings are highly relevant, as identifying viable and effective alternatives such as the combination of linalool with nematophagous fungi represents a promising advance toward the development of new strategies for helminth control.

The groups treated only with conidia G3 (10,000) and G4 (100,000) showed lower inhibitory effects on L3 larvae after 24 h, with reductions of 16.7% and 45.2%, respectively, reaching a maximum larval reduction of approximately 80% after 14 days of interaction. These findings reinforce that combining biological agents is advantageous and can be explored in integrated control programs to reduce the use of chemical products, the selection pressure for anthelmintic resistance, and therapeutic failures resulting from the use of a single agent, thereby providing a more effective and sustainable approach to animal production (Kaplan et al. 2020; Štrbac et al. 2023).

Thus, the combination of linalool with the fungus *A. cladodes* demonstrated promising anthelmintic activity, highlighting the potential of this association as a sustainable and innovative alternative in the integrated management of helminth infections in sheep. Moreover, this strategy may help reduce the excessive use of chemical anthelmintics and delay the progression of anthelmintic resistance. However, further in vivo studies are needed to better understand the synergy between these agents and to validate their efficacy and safety in parasite control.

## Data Availability

The datasets generated during and/or analyzed during the current study are available from the corresponding author on reasonable request.

## References

[CR1] Adeyinka A, Muco E, Regina AC, Pierre L (2023) Organophosphates. StatPearls. StatPearls Publishing, Treasure Island (FL) 29763035

[CR2] Aguiar AARM et al (2025) In vitro efficacy of the monoterpene linalool isolated or combined with the nematophagous fungus *Duddingtonia flagrans* in the control of sheep gastrointestinal nematodes. Microbiol Res 16(1). 10.3390/microbiolres1601000

[CR3] Alvares FBV et al (2025) Acaricidal efficacy of the monoterpene linalool against the cattle tick *Rhipicephalus microplus* and its synergistic potential with cypermethrin. Exp Appl Acarol 94:45. 10.1007/s10493-025-01011-z 40097703 10.1007/s10493-025-01011-z

[CR4] Burke JM, Miller JE (2020) Sustainable approaches to parasite control in ruminant livestock. Vet Clin N Am Food Anim Pract 36:89–107. 10.1016/j.cvfa.2019.11.007 10.1016/j.cvfa.2019.11.007 32029191

[CR5] Castagna F et al (2022) Potential new therapeutic approaches based on *Punica granatum* fruits compared to synthetic anthelmintics for the sustainable control of gastrointestinal nematodes in sheep. Animals 12:2883. 10.3390/ani12202883 36290268 10.3390/ani12202883 PMC9598348

[CR6] Costa VMM, Simões SVD, Riet-Correa F (2011) Gastro-intestinal nematodes control in goats and sheep in the semiarid region of northeastern Brazil. Pesq Vet Bras 31:65–71. 10.1590/S0100-736X2011000100010

[CR7] Ferreira LE et al (2013) *In vitro* anthelmintic activity of aqueous leaf extract of *Annona muricata* L. (Annonaceae) against *Haemonchus contortus* from sheep. Exp Parasitol 134:327–332. 10.1016/j.exppara.2013.03.032 23583362 10.1016/j.exppara.2013.03.032

[CR8] Gilleard JS et al (2021) A journey through 50 years of research relevant to the control of gastrointestinal nematodes in ruminant livestock and thoughts on future directions. Int J Parasitol 51(13–14):1133–1151. 10.1016/j.ijpara.2021.10.007 34774857 10.1016/j.ijpara.2021.10.007

[CR9] Gordon HM, Whitlock HV (1939) A new technique for counting nematode eggs in sheep faeces. J Counc Sci Ind Res 12:50–52

[CR10] Helal MA et al (2020) Nematocidal effects of a coriander essential oil and five pure principles on the infective larvae of major ovine gastrointestinal nematodes *in vitro*. Pathogens 9:740. 10.3390/pathogens9090740 32916863 10.3390/pathogens9090740 PMC7558654

[CR11] Helal MA et al (2022) Microfluidic based formulation of essential oils-loaded chitosan coated PLGA particles enhances their bioavailability and nematocidal activity. Pharmaceutics 14, 2030. 10.3390/pharmaceutics14102030 10.3390/pharmaceutics14102030 PMC9608619 36297465

[CR12] Kaplan RM (2020) Biology, epidemiology, diagnosis and management of anthelmintic resistance in gastrointestinal nematodes of livestock. Vet Clin N Am Food Anim Pract 36:17–30. 10.1016/j.cvfa.2019.12.001 10.1016/j.cvfa.2019.12.001 32029182

[CR14] Katiki LM et al (2017) Synergistic interaction of ten essential oils against *Haemonchus contortus in vitro*. Vet Parasitol 243:47–51. 10.1016/j.vetpar.2017.06.008 28807309 10.1016/j.vetpar.2017.06.008

[CR13] Katiki LM et al (2019) Evaluation of encapsulated anethole and carvone in lambs artificially- and naturally-infected with *Haemonchus contortus*. Exp Parasitol 197:36–42. 10.1016/j.exppara.2019.01.002 30633915 10.1016/j.exppara.2019.01.002

[CR15] Li S, Wang D, Gong J, Zhang Y (2022) Individual and combined application of nematophagous fungi as biological control agents against gastrointestinal nematodes in domestic animals. Pathogens 11(2):172. 10.3390/pathogens11020172 35215117 10.3390/pathogens11020172 PMC8879429

[CR16] Li X et al (2023) Revealing the mechanisms for linalool antifungal activity against *Fusarium oxysporum* and its efficient control of Fusarium wilt in tomato plants. Int J Mol Sci 24:458. 10.3390/ijms24010458 10.3390/ijms24010458 PMC9820380 36613902

[CR17] López MD, Pascual-Villalobos MJ (2010) Mode of inhibition of acetylcholinesterase by monoterpenoids and implications for pest control. Ind Crops Prod 31:284–288. 10.1016/j.indcrop.2009.11.005

[CR18] Luns FD et al (2018) Coadministration of nematophagous fungi for biological control over nematodes in bovine in the South-Eastern Brazil. Biomed Res Int 2018:2934674. 10.1155/2018/2934674 10.1155/2018/2934674 PMC5892246 29780820

[CR19] Maqbool I et al (2017) Integrated parasite management with special reference to gastrointestinal nematodes. J Parasit Dis 41:1–8. 10.1007/s12639-016-0765-6 28316380 10.1007/s12639-016-0765-6 PMC5339188

[CR20] Medeiros CIS et al (2022) Antifungal activity of linalool against fluconazole-resistant clinical strains of vulvovaginal *Candida albicans* and its predictive mechanism of action. Braz J Med Biol Res 55:e11831. 10.1590/1414-431X2022e11831 35976268 10.1590/1414-431X2022e11831 PMC9377531

[CR21] Mendes LQ et al (2023) Efficacy of *Duddingtonia flagrans* (Bioverm^®^) on the biological control of buffalo gastrointestinal nematodes. Exp Parasitol 253:108592. 10.1016/j.exppara.2023.108592 37549824 10.1016/j.exppara.2023.108592

[CR22] Nadeem M et al (2013) Nutritional and medicinal aspects of coriander (*Coriandrum sativum* L). Br Food J 115:743–755. 10.1108/00070701311331526

[CR23] Oliveira IC et al (2018) Using the fungus *Arthrobotrys cladodes* var. *macroides* as a sustainable strategy to reduce numbers of infective larvae of bovine gastrointestinal parasitic nematodes. J Invertebr Pathol 158:46–51. 10.1016/j.jip.2018.09.004 30240583 10.1016/j.jip.2018.09.004

[CR25] Ribeiro WLC, Vilela VLR (2023) Editorial: Alternatives for the control of parasites to promote sustainable livestock. Front Vet Sci 9:1097432. 10.3389/fvets.2022.1097432 36704708 10.3389/fvets.2022.1097432 PMC9872101

[CR24] Roberts FHS, O’Sullivan JP (1950) Methods of egg counts and larval cultures for Strongyles infesting the gastrointestinal tract of cattle. Aust J Agric Res 1:99–102. 10.1071/AR9500099

[CR27] Rodrigues JA et al (2020) Predatory effects of the fungus *Arthrobotrys cladodes* on sheep gastrointestinal nematodes. Biocontrol Sci Technol 30(8):830–839. 10.1080/09583157.2020.1775176

[CR26] Rodrigues JA et al (2022) Control of sheep gastrointestinal nematodes on pasture in the tropical semiarid region of Brazil, using Bioverm^®^ (*Duddingtonia flagrans*). Trop Anim Health Prod 54:179. 10.1007/s11250-022-03181-z 35511381 10.1007/s11250-022-03181-z

[CR28] Saeed F et al (2023) Lavender essential oil: nutritional, compositional, and therapeutic insights. In: Nayik GA, Ansari MJ (eds) Essential Oils: extraction, characterization and applications. Elsevier, Amsterdam, pp 85–101

[CR29] Sebai E et al (2022) Assessment of anthelmintic potentials of *Myrtus communis* against *Haemonchus contortus* and *Heligmosomoides polygyrus*. Exp Parasitol 312:109835. 10.1016/j.exppara.2022.108320 10.1016/j.exppara.2022.108320 35779645

[CR30] Simões SVD, Raimondo RFS, Correa BR (2022) Parasitoses gastrintestinais de pequenos ruminantes: os desafios do controle. RBB 2(2). 10.70061/2763-955X.2024.001

[CR31] Štrbac F et al (2023) *In vitro* and *in vivo* anthelmintic efficacy of peppermint (*Mentha x piperita* L.) essential oil against gastrointestinal nematodes of sheep. Front Vet Sci 10:1232570. 10.3389/fvets.2023.1232570 37662995 10.3389/fvets.2023.1232570 PMC10472939

[CR32] Taylor MA, Coop R, Wall RL (2017) Parasitologia Veterinária. 4ª ed. Rio de Janeiro: Guanabara Koogan

[CR34] Vieira ÍS, Oliveira IC, Campos AK, Araújo JV (2019) Association and predatory capacity of fungi *Pochonia chlamydosporia* and *Arthrobotrys cladodes* in the biological control of parasitic helminths of bovines. Parasitology 146(10):1347–1351. 10.1017/S003118201900060X 31148530 10.1017/S003118201900060X

[CR33] Vieira ÍS, Oliveira IC, Campos AK, Araújo JV (2020a) *Arthrobotrys cladodes* and *Pochonia chlamydosporia*: Nematicidal effects of single and combined use after passage through cattle gastrointestinal tract. Exp Parasitol 218:108005. 10.1016/j.exppara.2020.108005 32971132 10.1016/j.exppara.2020.108005

[CR35] Vieira ÍS, Oliveira IC, Freitas SG, Campos AK, Araújo JV (2020b) *Arthrobotrys cladodes* and *Pochonia chlamydosporia* in the biological control of nematodiosis in extensive bovine production system. Parasitology 147(6):699–705. 10.1017/S0031182020000098 32008588 10.1017/S0031182020000098 PMC10317615

[CR36] Vilela VLR et al (2018) Control of sheep gastrointestinal nematodes using the combination of *Duddingtonia flagrans* and Levamisole Hydrochloride 5%. Rev Bras Parasitol Vet 27(1):2–6. 10.1590/s1984-29612018008 10.1590/S1984-296120180011 29641796

[CR37] Vilela VLR et al (2020) Use of *Duddingtonia flagrans* in the control of gastrointestinal nematodes of feedlot goats. Semin Ciênc Agrár 41:915–924. 10.5433/1679-0359.2020v41n3p915

[CR38] Willcox HP, Coura JR (1989) Nova concepção para o método de Baermann-Moraes-Coutinho na pesquisa de larvas de nematódeos. Mem Inst Oswaldo Cruz 84:563–565. 10.1590/S0074-02761989000400015 2487451 10.1590/s0074-02761989000400015

[CR39] Zajíčková M et al (2020) Anthelmintics in the future: current trends in the discovery and development of new drugs against gastrointestinal nematodes. Drug Discov Today 25(2):430–437. 10.1016/j.drudis.2019.12.007 31883953 10.1016/j.drudis.2019.12.007

